# The Central Illinois Medical Society

**Published:** 1873-02

**Authors:** W. G. Cochran

**Affiliations:** Sec’y.


### *The Central Illinois Medical Society*

Met in regular session in Bloomington, January 14, 1873. Society was called to order at 10 A. M. by Dr. J. Wright, President.

On motion, the reading of the minutes of the preceding meeting was postponed till the afternoon session.

On motion of Dr. Laughlin the regular order of business was suspended and the society proceeded to the election of the following members, recommended by Dr. R. G. Laughlin:

T. F. Worrell, M.D.; J. L. White, M.D.; D. L. Crist, M.D.; D. C. Moore, M.D.; H. C. Luce, M.D.; N. B. Cole, M.D., of Bloomington, and W. L. Pollock, M.D., of Heyworth.

On motion, adjourned till two P. M.

AFTERNOON SESSION.

Called to order by President Wright.

Minutes of previous meeting read and approved.

On recommendation of Dr. Laughlin, L. Asire, M.D., of Bloomington, and J. Little, M.D., of LeRoy, were elected members of the society.

On motion, Dr. J. J. Starkey, of Louisville Medical College, was invited to participate in the proceedings of the society.

Dr. W. Hill, of committee on surgery, made a valuable and very interesting report on lithotomy, criticising very severely some of the modern modes of operating.

Recommended by Dr. Tenney, Dr. Lee Smith, of Rush Medical College, was elected a member of the society.

Dr. S. H. Birney, chairman of committee on obstetrics and diseases of females, read a very interesting essay on diseases of females. The production was a valuable one, and of great credit to its author.

On motion, Dr. Hill and Dr. Birney were requested to furnish copies of the reports for publication in the Chicago Medical Journal.

On motion of Dr. Laughlin, the publishing committee were instructed to have one hundred copies of the constitution and by-laws printed.

*Delegates to State Medical Society.*—Drs. S. H. Birney, W. G. Cochran, C. T. Orner, and W. L. Pollock.

*Delegates to American Medical Association.*—Drs. H. C. Luce, C. Goodbrake, S. H. Birney, J. Little, G. W. Barton, John Clouser, and W. Hill.

On motion, adjourned till seven P. M.

EVENING SESSION.

Called to order by Dr. Wright, President.

Dr. Laughlin, chairman of the committee on practical medicine, made a practical and very interesting report on the diseases prevalent in this country since the last meeting of the society. The report enlisted a lively discussion, and the doctor was requested to furnish copies for publication in the Chicago Medical Journal.

Dr. J. L. White, of Bloomington, delivered a very interesting address. Although prepared for the public it was very interesting to the society, and a vote of thanks was tendered the doctor for his valuable production.

The President announced the following special committees:

*Surgery.*—Drs. H. C. Luce, C. Goodbrake, and S. H. Birney.

*Practical Medicine.*—Drs. J. T. Pearman, Z. H. Madden, and G. W. Barton.

*Obstetrics and Diseases of Females.*—Drs. M. S. Brown, A. P. Tenney, and J. H. Potter.

*Pathology*—Dr. C. T. Orner.

*Microscopy.*—Dr. R. D. Bradley.

*Eye and Ear.*—Drs. N. B. Dole, H. C. Howard, and R. H. Huddleton.

*Committee of Arrangements.*—Drs. S. H. Birney, J. T. Pearman, and H. C. Howard.

Very interesting verbal reports were made by Drs. Worrell, Orner, and others.

The following resolution was unanimously adopted:

*Resolved*, That the thanks of the society are due to the committee of arrangements, the physicians of the city, the authorities of the county, and the trustees of the First Methodist Church, for kind care and hospitalities shown us while meeting within their midst.

On motion, the Secretary was instructed to furnish copies of the proceedings for publication in the county papers.

On motion, adjourned to meet in Champaign the second Tuesday in June, 1873.

Notwithstanding this was the first meeting of the society since its organization, it was well attended and the greatest interest manifested. We expect, however, to have a still more interesting time at our next meeting in Champaign, where we hope to meet all the physicians of the central portion of the State, and *every one* surcharged with "medical news."

W. G. COCHRAN, *Sec'y.*

